# The correlation between insulin resistance and blood lipids in children

**DOI:** 10.5937/jomb0-48461

**Published:** 2024-11-16

**Authors:** Li Jiang, Lulian Xu, Yang Lu, Xu Xu

**Affiliations:** 1 WuXi Children's Hospital, Department of Endocrine, Wuxi, Jiangsu Province, China

**Keywords:** children with simple obesity, insulin resistance, blood lipids, high-intensity interval training, deca sa jednostavnom gojaznošću, insulinska rezistencija, lipidi u krvi, intervalni trening visokog intenziteta

## Abstract

**Background:**

This work focused on the correlation between insulin resistance (IR) and blood lipids (BL) in children with simple obesity, as well as the intervention effects of high-intensity interval training (HIIT) in weight loss in children.

**Methods:**

80 children aged 6 to 17 years with simple obesity were selected from our hospital and randomly grouped into two groups. Children in the control (Ctrl) group underwent traditional moderate-intensity continuous training (MICT), while those in the other group received HIIT (HIIT group). After four weeks, body composition-related indicators, BL levels, and IR were measured.

**Results:**

After exercise, children in both groups possessed obvious reductions in body mass index (BMI), body fat percentage (BFP), triglyceride (TG), and homeostasis model assessment of IR (HOMA-IR), demonstrating remarkable differences in contrast to those after intervention (P<0.05). The HIIT group also exhibited considerable differences in waist circumference (WC), total cholesterol (TC), high-density lipoprotein cholesterol (HDL-C), and low-density lipoprotein cholesterol (LDL-C) (P<0.05). After exercise, the HIIT group demonstrated more observable BMI, WC, TG, and HOMA-IR reductions, showing great differences with the Ctrl group (P<0.05). HOMA-IR exhibited positive correlations with TC, TG, and HDL-C but a negative one with LDL-C (P<0.05).

**Conclusions:**

HIIT improved the body composition and BL levels in children with simple obesity, downregulated HOMA-IR, and positively impacted their health status. Furthermore, IR was associated with BL-relevant indicators in children with simple obesity.

## Abstract (sr)

**Uvod:**

Ovaj rad se fokusirao na korelaciju između insulinske rezistencije (IR) i lipida u krvi (BL) kod dece sa jednostavnom gojaznošću, kao i na interventne efekte intervalnog treninga visokog intenziteta (HIIT) na gubitku težine kod dece.

**Metode:**

Iz naše bolnice je odabrano 80 dece uzrasta od 6 do 17 godina sa jednostavnom gojaznošću i nasumično grupisano u dve grupe. Deca u kontrolnoj (Ctrl) grupi su podvrgnuta tradicionalnom kontinuiranom treningu umerenog intenziteta (MICT), dok su deca u drugoj grupi bila podvrgnuta HIIT (HIIT grupa). Posle četiri nedelje, mereni su indikatori u vezi sa telesnom kompozicijom, nivoi BL i IR.

**Rezultati:**

Nakon vežbanja, deca u obe grupe su imala očigledna smanjenja indeksa telesne mase (BMI), procenta telesne masti (BFP), triglicerida (TG) i procene modela homeostaze IR (HOMA-IR), pokazujući značajne razlike u odnosu na one posle intervencije (P<0,05). HIIT grupa je takođe pokazala značajne razlike u obimu struka (WC), ukupnom holesterolu (TC), holesterolu lipoproteina visoke gustine (HDL-C) i holesterolu lipoproteina niske gustine (LDL-C) (P<0,05). Nakon vežbanja, HIIT grupa je pokazala uočljivije smanjenje BMI, WC, TG i HOA-IR, pokazujući velike razlike u poređenju sa Ctrl grupom (P<0,05). HOMA-IR je pokazao pozitivnu korelaciju sa TC, TG i HDLC, ali negativnu sa LDL-C (P<0,05).

**Zaključak:**

Intervalnog trening visokog intenziteta (HIIT) je poboljšao telesnu kompoziciju i nivoe BL kod dece sa jednostavnom gojaznošću, smanjio HOMA-IR i pozitivno uticao na njihov zdravstveni status. Štaviše, IR je bio povezan sa indikatorima relevantnim za BL kod dece sa jednostavnom gojaznošću.

## Introduction

### Blood lipids (BL) and insulin resistance (IR) of children with simple obesity

With improvements in economic status, significant shifts in dietary patterns and lifestyle habits have occurred, contributing to the rising prevalence of obesity in society, particularly among children. This trend is fueled by the consumption of high-energy foods and a decline in physical activity. Simple obesity refers to non-pathological excessive fat accumulation or abnormal fat distribution in the body, resulting from an imbalance between energy intake and expenditure. It constitutes over 95% of the obese population and typically lacks apparent pathological causes, often manifesting with metabolic disturbances, including insulin resistance (IR) and abnormal baseline (BL) levels.

IR involves a weakened cellular response to insulin, leading to impaired biological effects. Multiple mechanisms contribute to IR, encompassing abnormal insulin receptor signalling and dysregulation of downstream signalling molecules. In children with simple obesity, IR commonly coexists with disturbances in lipid metabolism, affecting adipose tissue, liver, and muscle lipid metabolism, and is closely linked with abnormal BL levels. Studies have demonstrated that children with simple obesity often exhibit abnormal BL profiles, characterised by elevated total cholesterol (TC), low-density lipoprotein cholesterol (LDL-C), and triglycerides (TG), alongside reduced high-density lipoprotein cholesterol (HDL-C). This dysregulated BL profile poses a risk factor for cardiovascular diseases. Without timely intervention, childhood simple obesity may precipitate various health issues, including an elevated risk of type 2 diabetes, hypertension, and cardiovascular diseases in adulthood. Excessive weight burden may also impede skeletal development, increase susceptibility to skeletal injuries, and adversely affect children’s mental health.

### Application of high-intensity interval training (HIIT) in weight loss of children

Treating simple obesity involves multiple aspects, including dietary management, exercise, and behaviour modification. Exercise, especially aerobic exercise, is considered the most effective means of improving simple obesity and reducing the risk of cardiovascular diseases. Some studies have suggested that exercise intensity has a greater impact on weight loss in children than exercise duration [1] [2] [3] [4] [5]. HIIT is a training method involving alternating high-intensity exercise and short recovery periods. It is believed to enhance cardiovascular endurance, increase muscle strength, and improve metabolic function. Relevant studies have shown that after 8 weeks of HIIT in overweight and obese children, their weight, body fat, and waist circumference (WC) sharply decreased. Compared to traditional aerobic exercise, HIIT offers time efficiency. Due to its shorter training time, children are more likely to adhere to and incorporate it into their daily lives. This is crucial for increasing the exercise participation of children and the success rate of weight loss. However, applying HIIT to children requires careful consideration of safety issues. Due to its high-intensity nature, such training needs to be adjusted according to children’s age, physical fitness level, and individual differences. Proper training arrangements and mature guidance are essential for ensuring safety and effectiveness [6] [7] [8] [9] [10].

Although there have been many studies demonstrating the effectiveness of HIIT for weight loss in various populations, there is still limited research on the use of HIIT in children with simple obesity. This work focused on the correlation between IR and BL in 80 children with simple obesity from our hospital. Additionally, it evaluated the intervention effects of HIIT on these children by assessing various relevant indicators. The primary goal was to explore the relationship between IR and BL in this specific group of children with simple obesity and to assess the potential benefits of HIIT as an intervention method.

## Materials and methods

### Study design and population

This work involved investigating 80 children with simple obesity from April 2020 to April 2023. Criteria for enrolling and excluding the children were presented in Table 1 and Table 2, respectively. The children with simple obesity were randomly rolled into two groups, namely, the Ctrl group and the HIIT group. All children with simple obesity were aged between 6 and 17 years. Before the study began, the children with simple obesity and their legal guardians were informed of the details of the experiment and obtained informed consent. The Ethics Committee of our hospital approved the study with the approval number 2020PN017.

**Table 1:** Criteria for children enrollment.

No.	Inclusion criteria:
1	Satisfying the diagnostic criteria<br>for simple obesity
2	Age between 6 to 17 years
3	No regular physical exercise in the<br>month prior to the study
4	Voluntary participation in this study, with<br>informed consent from their guardians

**Table 2:** Criteria to exclude the children from this work.

No.	Exclusion criteria:
1	With secondary obesity caused by congenital diseases, endocrine disorders, etc
2	With severe liver or kidney dysfunction
3	With heart disease or musculoskeletal disorders
4	Taking medications that affect metabolism
5	With poor compliance or unable to complete follow-up

Simple randomisation procedures were employed to minimise bias, ensuring that each child had an equal chance of being assigned to either group. A simple randomisation method was employed for randomisation. This involved assigning each eligible child with simple obesity a unique identifier. These identifiers were then placed in a container using a computer-generated randomisation list. A researcher who was not involved in the enrollment process randomly selected identifiers from the container one at a time, assigning each selected child to either the Control (Ctrl) group or the High-Intensity Interval Training (HIIT) group based on the group assignment associated with the identifier.

This process ensured that each child had an equal chance of being assigned to either group, minimising selection bias and ensuring a fair distribution of participants across the two groups. Additionally, to maintain blinding and prevent potential bias, the researcher responsible for randomisation was kept unaware of the characteristics or preferences of the participants.

### Treatment operations

**Figure 1:**
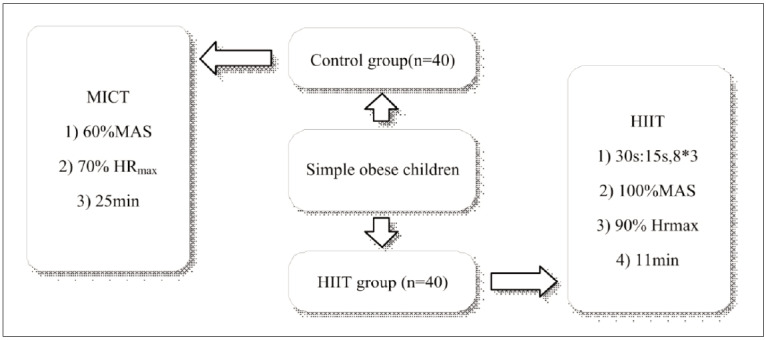
Training schemes for children in different groups.

All children with simple obesity received running interventions at an outdoor track and field. A two-week adaptive training was arranged based on the physical condition of the children. The formal training sessions were held every other day, four days a week (Monday, Wednesday, Friday, and Sunday). The training plan was illustrated in Figure 1. Children in the Ctrl group underwent moderate-intensity continuous training (MICT) with a load intensity of 60% of the maximum aerobic speed (MAS), maintaining their heart rate (HR) at approximately 70% of maximum heart rate (HRmax) for a continuous duration of about 25 minutes. In contrast, those in the HIIT group performed a warm-up jog followed by muscle activation. The interval training consisted of running at 100% of MAS for 30 seconds, followed by a 15-second rest period. Each set was repeated 8 times, with 3 sets in total. The HR was maintained at around 90% of HRmax during the HIIT sessions. The formal training lasted 4 weeks for all children in the Ctrl and HIIT groups.

### Observation indicators

### Body composition

In this study, we assessed the body composition of children with simple obesity using the bioelectrical impedance method. Data, including BMI, body fat percentage (BFP), and waist circumference (WC), were recorded using the BC-601 Tanita Body Composition Analyzer. We followed the instructions provided by the body composition analyser for data collection.

Upon inputting basic information such as height, age, and gender of the children being tested, individual profiles were created. The children were instructed to stand barefoot on the foot electrodes of the instrument while wearing close-fitting and lightweight clothing. Holding the hand electrodes with both hands, they were asked to keep their arms apart and not in contact with their bodies. Throughout the measurement, participants remained still and refrained from movement.

A painless electric current was administered to measure the body’s impedance to the current, enabling the acquisition of data on body mass and various body composition parameters.

### Blood indicators

Before collecting blood samples, enrolled children were required to fast for 10 hours. Approximately 5 mL of blood was drawn from the left arm vein and allowed to stand at room temperature for 30 minutes. Subsequently, the blood samples underwent centrifugation at 2,000 revolutions per minute (rpm) for 10 minutes using a centrifuge with a radius of 12 cm. Following centrifugation, the upper layer of serum was carefully collected and stored at -80°C for further analysis.

Using an automated biochemical analyser, the serum samples were subjected to the enzymatic colourimetric method to examine total cholesterol (TC), triglycerides (TG), high-density lipoprotein cholesterol (HDL-C), and low-density lipoprotein cholesterol (LDL-C). Additionally, the fasting blood glucose (FBG) level and insulin level were determined using the glucose oxidase and radioimmunoassay methods, respectively.

Furthermore, the Homeostatic Model Assessment of Insulin Resistance (HOMA-IR) was employed to assess the degree of insulin resistance. HOMA-IR was calculated using the formula: (fasting insulin × FBG)/22.5.

### Methods for statistical analysis

Data were statistically analysed using SPSS 20.0, and the results were presented as mean ± standard deviation. Paired t-tests were arranged to compare the changes in various indicators before and after the intervention in the same group. Meanwhile, independent sample t-tests were used to compare the two groups’ differences in different indicators. Additionally, Pearson rank correlation (PRC) analysis was utilised to assess the correlation among various variables. *P*<0.05 was considered statistically significant for all analyses.

## Results

The Ctrl group consisted of 22 boys and 18 girls, with an average age and body mass index (BMI) of (12.3±0.7) years old and (24.4±1.3) kg/m^2^, respectively. The HIIT group enrolled 20 boys and 20 girls, with an average age and BMI of (11.8±0.5) years old and (24.0±1.5) kg/m^2^, respectively. Statistical analysis on the composition of genders, ages, WC, BMIs, and maximum oxygen consumption (MOC) of children indicated a comparability between the Ctrl and HIIT groups (*P*>0.05).

### Changes in body composition of children before and after exercise

**Figure 2:**
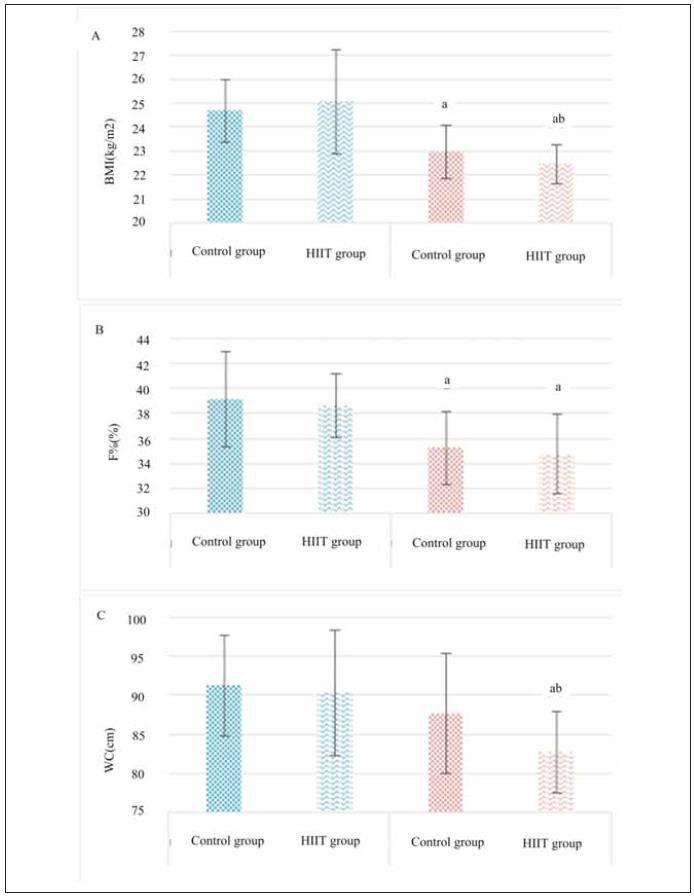
Changes in indicators of body composition of children before and after different exercises. Note: a suggested a significant difference with P<0.05 to the pre-intervention value, while b suggested a significant difference with P<0.05 to the Ctrl group

The BMI, BFP, and WC data for children were recorded and compared before and after exercise, as illustrated in Figure 2. Following exercise, all children showed a reduction in BMI and BFP, with significant differences observed compared to pre-exercise values (*P*<0.05). Specifically, the HIIT group exhibited a significant decrease in BMI (*P*<0.05), while the difference in BFP was not significant (*P*>0.05). Additionally, compared to pre-exercise levels, children in the HIIT group significantly reduced WC (*P*<0.05). Furthermore, a noticeable difference in WC was observed between the HIIT and Ctrl groups, with a significant distinction noted (*P*<0.05). Table 3

**Table 3:** Baseline characteristics of included participants.

Variable	HIIT Group<br>N=40	Control Group<br>N=40	P-value
Age (years)	11.8±0.5	12.3 0.7	0.455
Gender<br>Male<br>Female	<br>20 (50.0%)<br>20 (50.0%)	<br>22 (55.0%)<br>18 (45.0%)	0.543
BMI (kg/m^2^)	24.0±1.5	24.4±1.3	0.892
WC (cm)	90.0±8.4	91.3±6.6	0.854
Body Fat (%)	38.6±2.5	39.1±3.9	0.376

### Changes in BL levels of children before and after exercise

**Figure 3:**
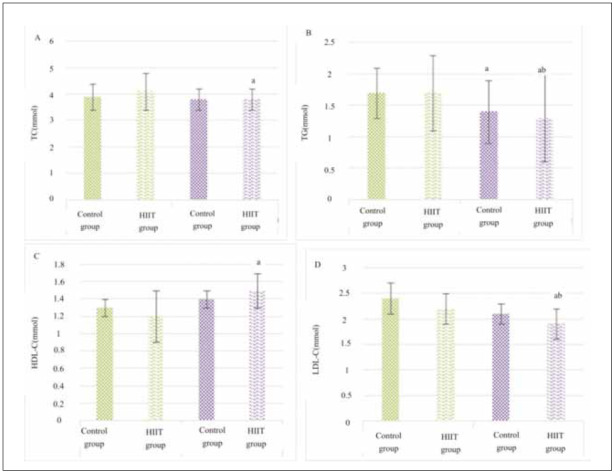
Changes in BL-related indicators for children before and after exercise. Note: a suggested a great difference with P<0.05 to the pre-intervention value, while b suggested an observable difference with P<0.05 to the Ctrl group.

TC, TG, HDL-C, and LDL-C levels in children were compared in Figure 3 before and after intervention. In the Ctrl group, although these indicators decreased after exercise, differences in TC, HDL-C, and LDL-C levels were not significant (*P*>0.05), with only TG showing a notable difference (*P*<0.05). In contrast, the HIIT group exhibited a significant decrease in serum TC, TG, HDL-C, and LDL-C levels after exercise (*P*<0.05). Furthermore, changes in TG and LDL-C levels were more pronounced in the HIIT group, showing significant differences compared to the Ctrl group (*P*<0.05).

### IR condition of children in various groups

**Figure 4:**
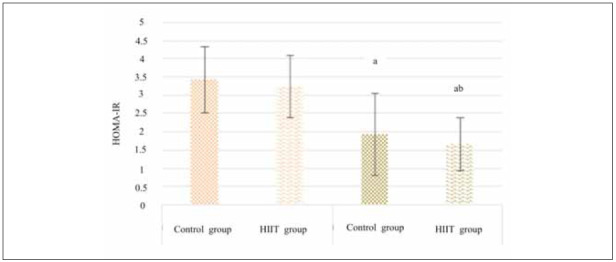
Changes in IR of children before and after different exercises. Note: a suggested a great difference with P<0.05 to the pre-intervention value, while b suggested an observable difference with P<0.05 to the Ctrl group.

Levels of FBG and insulin in children across different groups were measured before and after exercise, and their HOMA-IR values were calculated. Results depicted in Figure 4 indicated that, compared to pre-exercise conditions, children in all groups experienced decreased HOMA-IR, with significant differences observed (*P*<0.05). Particularly noteworthy was the HIIT group, which displayed a more pronounced reduction in HOMA-IR, demonstrating a significant difference compared to the Ctrl group (*P*<0.05).

### Correlation between IR and BL

Post-exercise TC, TG, and HDL-C in the HIIT group exhibited a positive correlation with HOMA-IR, whereas LDL-C showed a negative correlation with HOMA-IR (*P*<0.05) (Table 4).

**Table 4:** Correlations between various BL-relevant indicators and HOMA-IR.

	r	*P*
TC	0.623	<0.05
TG	0.582	<0.05
HDL-C	-0.564	<0.05
LDL-C	0.617	<0.05

## Discussion

### Blood glucose and BL metabolism of children with simple obesity

Simple obesity, a prevalent metabolic disorder in children, is closely associated with blood glucose and BL metabolism disruptions. Elevated body weight and fat accumulation induce insulin resistance, a hallmark of simple obesity. Insulin plays a pivotal role as a regulatory hormone, facilitating glucose uptake, utilisation, and storage. However, in the context of IR, cells exhibit reduced responsiveness to insulin, leading to inefficient glucose uptake and utilisation, consequently elevating blood glucose levels [11]. Notably, IR impacts glucose metabolism and has significant implications for BL metabolism. Children with simple obesity often exhibit BL abnormalities, wherein IR prompts heightened release of fatty acids from adipose tissue into circulation while inhibiting their oxidation [12]. Consequently, plasma levels of free fatty acids increase alongside heightened triglyceride synthesis, culminating in elevated plasma triglyceride concentrations [13]. Furthermore, IR influences lipoprotein metabolism, downregulating HDL-C and upregulating LDL-C and TC, thereby augmenting the risk of atherosclerosis and cardiovascular diseases [14] [15].

Unhealthy dietary habits constitute a significant contributing factor to simple childhood obesity. Diets rich in energy, fats, and sugars exacerbate this condition by promoting fat deposition and weight gain. Moreover, high-sugar diets can precipitate substantial fluctuations in blood glucose and insulin levels, exacerbating IR and disrupting blood glucose metabolism, thereby imposing strain on the kidneys [16]. Concurrently, insufficient physical activity represents another pivotal factor influencing abnormal blood glucose and BL metabolism in children with simple obesity. Physical exercise not only aids in expending excess energy and reducing fat accumulation but also enhances insulin sensitivity and improves blood glucose and BL metabolism [17] [18]. Specifically, aerobic exercise facilitates glucose uptake and utilisation, thereby mitigating IR, while resistance training fosters increased muscle mass and metabolic rate, further enhancing blood glucose and BL metabolism [19]. Extensive research has explored the comparative effects of aerobic exercise, resistance training, and a combination of both on blood glucose and BL metabolism. Findings suggest that combined training may promote fatty acid oxidation more effectively and impede cholesterol and fatty acid synthesis via the AMPK pathway [20] [21].

### Impacts of HIIT on body composition of children with simple obesity

HIIT, which integrates high-intensity exercise segments with recovery or low-intensity exercise segments, enhances cardiovascular endurance, bolsters muscle strength, and facilitates fat burning. Compared to traditional continuous aerobic exercises, HIIT offers the advantage of achieving higher exercise intensity and energy expenditure within a relatively brief timeframe. Moreover, HIIT can be adapted to diverse forms of exercise, including running, cycling, swimming, or aerobics. The typical training regimen involves alternating between high-intensity and low intensity exercise segments, thereby fostering muscle strength and endurance improvements, stimulating muscle growth and development, enhancing cardiac and pulmonary function, elevating maximum oxygen consumption, and enhancing overall physical performance [22] [23].

Furthermore, HIIT proves effective in promoting fat loss. Obesity is characterised by adverse alterations in body composition, marked by excessive fat tissue accumulation and relative reduction of muscle tissue. HIIT elevates the body’s metabolic rate, facilitating fat oxidation and breakdown, thereby promoting fat burning and reducing overall body fat content. HIIT also stimulates the release of endorphins and endogenous hormones, enhancing mental health and emotional well-being and promoting psychological wellness alongside physical functionality.

The results of this study indicate that children with simple obesity experienced reductions in BMI, body fat percentage (BFP), and WC following exercise, with significant differences observed in BMI and BFP between the control (Ctrl) and HIIT groups (*P*<0.05). Specifically, children in the HIIT group exhibited more pronounced decreases in BMI and WC, demonstrating significant disparities compared to those in the Ctrl group (*P*<0.05). However, the difference in BFP did not reach statistical significance (*P*>0.05). These findings suggest that HIIT may positively influence alterations in the body composition of children with simple obesity, particularly in reducing abdominal fat. This corroborates the findings of a study by Zhou et al. [24].

### Impacts of HIIT on BL and IR of children with simple obesity

Abnormal blood glucose and BL metabolism are prominent metabolic changes observed in individuals with obesity. The increase in fat cell count and accumulation of adipose tissue in obesity leads to heightened release of fatty acids, which disrupts insulin’s action on glucose regulation. Previous studies have reported altered hormones produced by fat cells in obese children, such as elevated leptin and reduced adiponectin levels, which may impact insulin signalling pathways and other physiological mechanisms. The deposition of free fatty acids in pancreatic and hepatic cells results in fat accumulation, affecting insulin synthesis and secretion by pancreatic β-cells, ultimately contributing to IR. IR refers to diminished cellular responsiveness to insulin, necessitating increased insulin levels to regulate normal blood glucose. Elevated blood glucose levels stimulate insulin secretion, promoting fatty acid synthesis and inhibiting lipolysis, further elevating BL levels. Lipid accumulation in tissues, particularly in muscles and the liver, disrupts insulin signalling, reduces cellular sensitivity to insulin, and perpetuates a cycle of worsening IR and metabolic disturbances associated with obesity [25] [26] [27] [28] [29].

The present study demonstrated that, compared to pre-exercise conditions, all BL indicators decreased in the control group, with only triglycerides showing a statistically significant difference. Both triglycerides and low-density lipoprotein cholesterol levels differed significantly between children in the control and HIIT groups. Following exercise, the Homeostatic Model Assessment of Insulin Resistance significantly decreased in all enrolled children, indicating an improvement in IR. Notably, this improvement in HOMA-IR was more pronounced in the HIIT group, consistent with previous research findings. However, while some BL indicators exhibited improvement, they did not reach statistical significance. This may be attributed to the relatively short exercise duration, resulting in less discernible effects, and the lack of detailed dietary planning and monitoring, which could have further influenced BL levels. Additionally, a positive correlation was observed between total cholesterol, triglycerides, high-density lipoprotein cholesterol, and HOMA-IR, while low-density lipoprotein cholesterol was negatively correlated with HOMA-IR, consistent with findings from previous studies.

The relatively short duration of the exercise intervention may have contributed to the observed lack of statistical significance in some baseline (BL) indicators. Moreover, the absence of detailed dietary planning and monitoring could have further confounded the effects on BL levels. These limitations suggest that the observed improvements, particularly in BL indicators that did not reach statistical significance, should be interpreted with caution. Furthermore, the short intervention duration and lack of dietary control may have limited the magnitude of the observed effects, underscoring the need for longer-term studies with more rigorous dietary protocols. This nuanced consideration highlights the importance of addressing these methodological limitations in future research to better elucidate the effects of exercise interventions on metabolic health in children.

In conclusion, this study elucidates a correlation between baseline (BL) levels and insulin resistance (IR) in children with simple obesity. High-intensity interval Training (HIIT) demonstrates the potential to positively impact body composition and serum lipid profiles and improve IR, thereby enhancing the overall health of children with simple obesity. However, the study has limitations, notably the short intervention duration and lack of dietary control. Thus, further research is warranted to address these limitations and explore alternative interventions, including different dietary approaches and HIIT exercise programs, to optimise the improvement of health-related indicators in this population. This underscores the importance of ongoing investigation to refine strategies for managing obesity-related metabolic complications in children.

## Dodatak

### Funding

The research is supported by: Study on the intervene effect of a comprehensive platform based on weight management on children at high risk of obesity and diabetes (No.: FYKY202206).

### Conflict of interest statement

All the authors declare that they have no conflict of interest in this work.
